# Impact of Body Weight Loss During Preoperative Chemoradiotherapy on Prognosis of Patients With Lower Rectal Cancer

**DOI:** 10.1002/ags3.70146

**Published:** 2025-12-15

**Authors:** Shinya Abe, Hiroaki Nozawa, Kazuhito Sasaki, Koji Murono, Shigenobu Emoto, Yuichiro Yokoyama, Yuzo Nagai, Yuzo Harada, Takahide Shinagawa, Soichiro Ishihara

**Affiliations:** ^1^ Department of Surgical Oncology, Graduate School of Medicine The University of Tokyo Bunkyo‐ku Tokyo Japan; ^2^ Department of Surgery The Fraternity Memorial Hospital Sumida‐ku Tokyo Japan

**Keywords:** body weight loss, chemoradiotherapy, outcome, rectal cancer

## Abstract

**Background:**

Preoperative chemoradiotherapy is the standard treatment for locally advanced rectal cancer (LARC), but may cause body composition changes. The prognostic impact of these changes remains unclear. The present study investigated the relationship between changes in body weight and composition during chemoradiotherapy, such as the psoas muscle mass index, visceral fat index, and subcutaneous fat index (SFI) obtained from computed tomography images, and long‐term outcomes in LARC patients.

**Methods:**

This retrospective study included 343 patients with LARC treated with preoperative chemoradiotherapy followed by curative resection between 2005 and 2023 at our hospital. Changes in body composition during chemoradiotherapy were retrospectively assessed, and the relationship between each index and long‐term outcomes was examined.

**Results:**

Forty‐one patients had body weight loss ≥ 10% during chemoradiotherapy, which correlated with reductions in 5‐year overall survival (OS) (68.2% vs. 90.5%, *p* < 0.001) and disease‐free survival (DFS) (53.5% vs. 70.9%, *p* = 0.005) rates. SFI loss ≥ 22.0% was also associated with a lower 5‐year OS rate (74.3% vs. 88.3%, *p* < 0.001). A multivariate analysis identified body weight loss ≥ 10% (HR 2.98, *p* = 0.001) and SFI loss ≥ 22.0% (HR 2.84, *p* = 0.007) as independent predictors of worse OS. Body weight loss ≥ 10% was also independently associated with worse DFS (HR 1.79, *p* = 0.014). Pre‐chemoradiotherapy body mass index ≥ 25 kg/m^2^ and the cT4 factor had high odds ratios for body weight loss ≥ 10% during chemoradiotherapy.

**Conclusion:**

Body weight loss during preoperative chemoradiotherapy is an independent predictor of worse OS and DFS in LARC.

## Graphical abstract

The present study aimed to investigate the relationship between changes in body weight and composition during chemoradiotherapy, such as the psoas muscle mass index, visceral fat index, and subcutaneous fat index obtained from computed tomography images, and long‐term outcomes in rectal cancer patients. This retrospective study included 343 patients with rectal cancer treated with preoperative chemoradiotherapy followed by curative resection between 2005 and 2023 at our hospital. Body weight loss during preoperative chemoradiotherapy is an independent predictor of worse OS and DFS in rectal cancer.

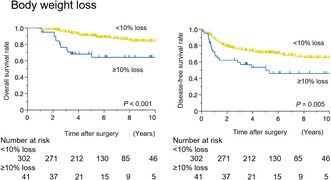

## 1 Introduction

Multimodal preoperative treatment for locally advanced rectal cancer (LARC) has resulted in a reduction in the risk of disease recurrence in the pelvis. Therefore, total mesorectal excision following chemoradiotherapy (CRT) is the standard treatment for patients with LARC. Although the majority of patients with LARC derive benefit from preoperative CRT, some develop adverse events during preoperative treatment, which might impact on long‐term outcomes [1]. Gastrointestinal toxicities may contribute to a poor nutritional status, leading to body weight loss and body composition changes during preoperative CRT [2], with the body mass index (BMI) being used to estimate body composition.

Computed tomography (CT) images have recently enabled objective measurements of the body composition of patients with cancer. Body composition obtained from CT at the third lumbar level, showing skeletal muscle and fat areas, is regarded as an essential biomarker that reflects inflammatory and nutritional statuses and is associated with various clinical cancer outcomes [3]. A recent systematic review and meta‐analysis demonstrated that skeletal muscle loss during neoadjuvant therapy was a prognostic indicator in patients with gastrointestinal cancers [4]. In addition, changes in adipose tissue during preoperative therapy have been associated with long‐term outcomes in ovarian cancers [5].

However, in the rectal cancer field, few studies have investigated the relationship between body composition changes during CRT and long‐term outcomes. Chung et al. [6] analyzed 93 LARC patients and indicated the potential of skeletal muscle loss as a predictor of overall survival (OS). Levolger et al. [7] examined 122 LARC patients and identified skeletal muscle loss as an independent predictor of disease‐free survival (DFS), but not OS. These two studies were relatively small and lacked a thorough assessment of changes in body composition, including fat volume, and the identification of parameters that are the most promising for predicting long‐term outcomes. Therefore, we herein investigated changes in body weight and composition during CRT to identify which parameters predict long‐term survival in patients with LARC.

## 2 Patients and Methods

### 2.1 Patients, Study Design, and Outcomes

We retrospectively evaluated 343 patients with clinical stage II‐III lower rectal adenocarcinoma treated with curative‐intent surgery following CRT at The University of Tokyo Hospital between April 2005 and March 2023. Patients with recurrent rectal cancer, with stage IV disease, with distant metastasis during preoperative chemoradiotherapy, and those who had not undergone rectal resection and requested non‐operative management were excluded. None of the patients included had undergone rectal resection due to their body condition (Figure S1). The pretreatment clinical stage was diagnosed using CT and magnetic resonance imaging. Pre‐CRT body weight measurements, blood sampling, and CT scans were performed 2–4 weeks before CRT, and post‐CRT body weight measurements, blood sampling, and CT scans were conducted 3–5 weeks after the completion of CRT. The present study was approved by the Institutional Ethics Committee of The University of Tokyo (No. 3252‐[15]) and informed consent was obtained through an opt‐out method.

### 2.2 Treatment

CRT consisted of long‐course radiotherapy (55 Gy/25 fractions for 5 weeks or 50.4 Gy/28 fractions for 6 weeks) and concurrent chemotherapy, mainly 5‐fluorouracil‐based oral administration, with or without CPT‐11 or oxaliplatin. Rectal surgery was typically performed 6–12 weeks after the completion of CRT. Lateral lymph node dissection was selectively performed on patients with swollen lateral lymph nodes with a longitudinal diameter ≥ 8 mm detected on CT before CRT [8]. All resected specimens were histopathologically analyzed. Pathological staging and tumor regression grading in the primary tumors were performed according to the Japanese Classification of Colorectal, Appendiceal, and Anal Carcinoma of the Japanese Society for Cancer of the Colon and Rectum [9]. Therefore, patients with pathologically positive lateral lymph nodes were classified into ypN‐stage ≥ 1. Adjuvant chemotherapy was considered for all patients according to the Japanese Society for Cancer of the Colon and Rectum guidelines [10].

### 2.3 Evaluation of Adverse Events

Patients were evaluated for adverse events during CRT and in the interval between CRT and surgery every 1–2 weeks according to the National Cancer Institute Common Terminology Criteria for Adverse Events (CTCAE), version 5. As previously described [11], body weight loss ≥ 10% after the completion of CRT was selected as the cut‐off for severe body weight loss according to CTCAE, version 5.

### 2.4 Patient Follow‐Up

Postoperative surveillance was generally performed for 5 years postoperatively. It consisted of laboratory tests, including carcinoembryonic antigen levels (every 3 months), chest and abdominal CT (every 6 months), and colonoscopy (every year) according to the Japanese Society for Cancer of the Colon and Rectum guidelines [10].

### 2.5 Assessment of Body Composition

Body composition was assessed in the present study using the following indices: the psoas muscle mass index (PMI), visceral fat index (VFI), and subcutaneous fat index (SFI). Body composition was assessed using pre‐CRT and post‐CRT CT images, which were extracted from multidetector CT datasets and imported into a three‐dimensional image analysis system (ZIOWORKSTATION 2; Ziosoft Inc., Tokyo, Japan). The psoas muscle area was evaluated based on Hounsfield unit (HU) thresholds of −29 to +150 at the third lumbar vertebra (L3) level using CT images [12]. Visceral fat and subcutaneous fat areas were evaluated based on HU thresholds of −200 to −50 HU at the L3 level [5]. These fat areas were also semiautomatically recognized and calculated. Body composition measurements were performed using established methodology [12] by a single author (SA). The psoas muscle area, visceral fat area, and subcutaneous fat area were normalized by patients' height squared (m^2^). Sarcopenia was defined according to the PMI cut‐off values for Asian adults (6.36 cm^2^/m^2^ for males and 3.92 cm^2^/m^2^ for females) [13]. Patients with pre‐ or post‐CRT sarcopenia were classified using CT images before and after CRT, respectively.

### 2.6 Statistical Analysis

Body weight, BMI, and L3 sectional body composition indices before and after CRT were compared using the paired *t*‐test. We calculated each patient's changes in BMI and body composition indices as follows: (x Post‐CRT − x Pre‐CRT)/x Pre‐CRT × 100. The cut‐off values for PMI, VFI, and SFI as indicators of severe skeletal muscle loss, visceral fat loss, and subcutaneous fat loss were set as the lowest quartile of changes in this cohort. Relationships among categorical and continuous variables and relevant outcome variables were assessed using the chi‐square and Mann–Whitney *U* tests. The trend of the patient's percentages under the median of body composition indices for body weight changes was assessed using the Cochran‐Armitage trend test. The Kaplan–Meier method with the Log‐rank test was used in OS, cancer‐specific survival (CSS), DFS, and recurrence‐free survival (RFS) analyses. In multiple comparisons adjustments for log‐rank tests, to address the issue of multiple comparisons, the Holm method was employed to control the Family‐Wise Error Rate. OS was defined as the interval between the date of rectal resection and death or the last follow‐up. CSS was defined as the interval between the date of rectal resection and disease‐specific death or the last follow‐up. DFS was defined as the interval between the date of rectal resection and death, recurrence, the detection of malignant disease, or the last follow‐up. RFS was defined as the interval between the date of rectal resection and death, recurrence, or the last follow‐up. A Cox proportional hazard regression was employed for a multivariable analysis of variables with *p* < 0.05 in the univariable analysis. Age‐ and sex‐adjusted odds ratios (OR) and 95% confidence intervals (CI) for the predictor of body weight loss ≥ 10% during CRT were calculated using logistic regression. A sensitivity analysis was performed based on the treatment strategy era due to the introduction of minimally invasive surgery and the CPT‐11 chemotherapy regimen for CRT in 2012 and 2018, respectively. All analyses were performed using JMP Pro 16.0 software (SAS Institute Inc., Cary, NC, USA). *p* < 0.05 was considered to be significant.

## 3 Results

### 3.1 Patient Characteristics

**FIGURE 1:**
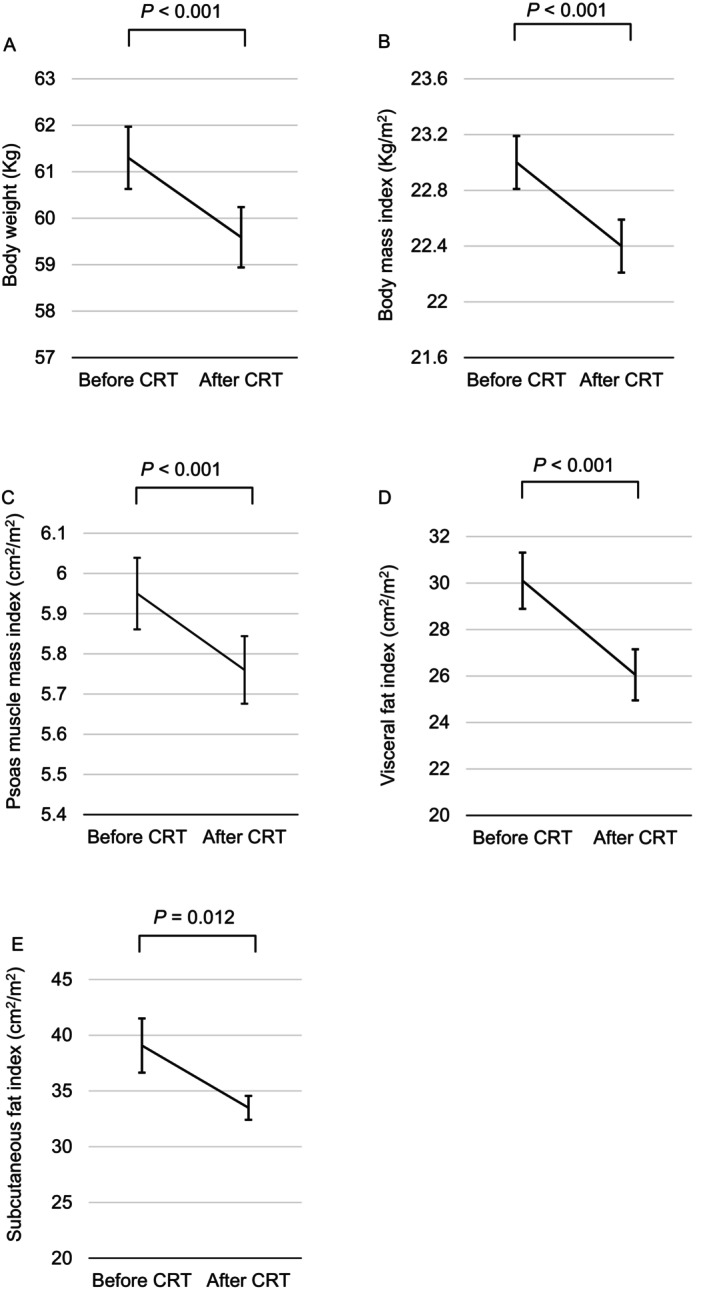
Comparison of body composition indices before and after CRT. (A) Body weight, (B) the body mass index, (C) psoas muscle mass index, (D) visceral fat index, and (E) subcutaneous fat index.

**FIGURE 2:**
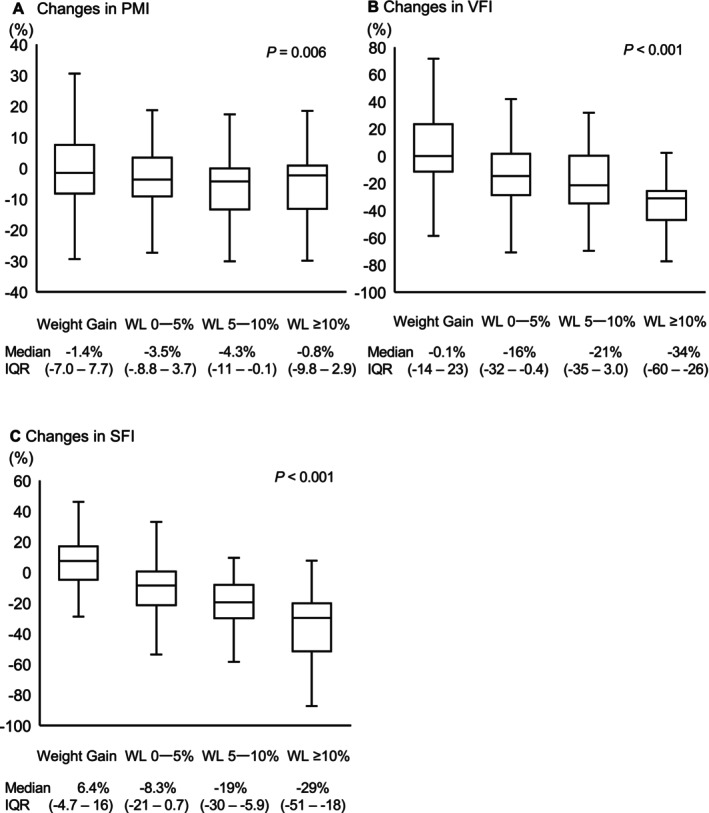
Distribution of percent changes in body composition according to body weight loss (WL). (A) The psoas muscle mass index, (B) visceral fat index, and (C) subcutaneous fat index. IQR, interquartile range.

The clinicopathological characteristics of 343 patients are summarized in Table 1. Median age was 64 years ([interquartile range] IQR: 56–71), and 219 patients (63.8%) were male. The median time to surgery from the completion of CRT was 57 days (IQR: 49–68). Most patients had cT3 or cT4 (98.5%), and 171 (49.9%) had clinical lymph node metastasis. The most commonly used chemotherapy regimen was tegafur/uracil plus leucovorin (74.3%). Regarding changes in body composition during CRT, median pre‐CRT body weight and BMI were 60.4 kg (IQR: 53.1–67.5) and 22.7 kg/m^2^ (IQR: 20.8–24.8), respectively. Median post‐CRT body weight and BMI were 59.0 kg (IQR: 50.8–66.0) and 22.0 kg/m^2^ (IQR: 20.3–24.1), respectively. These parameters significantly decreased during CRT (*p* < 0.001, *p* < 0.001, respectively; Figure 1A,B). We also observed significant changes in PMI, VFI, and SFI during CRT (*p* < 0.001, *p* < 0.001, *p* = 0.012, respectively; Figure 1C–E). Median percent changes from before to after CRT were −2.6% (IQR: −9.1%—4.1%) in PMI, −11.9% (IQR: −29.2%—6.3%) in VFI, and −7.3% (IQR: −22.0%—6.6%) in SFI. The cut‐off values for severe skeletal muscle loss, visceral fat loss, and subcutaneous fat loss were set at 9.1%, 29.2%, and 22.0%, respectively. We then examined the relationship between each body composition index and the degree of body weight loss (Figure 2). The rate of the reduction in PMI did not decrease further with weight loss, whereas those in VFI and SFI continued to decrease. Figure S2 shows the percentage of cases under the median of each body composition index in each group: 44%, 53%, 61%, and 42%, respectively, for PMI loss (*p* = 0.06), 20%, 54%, 75%, and 87%, respectively, for VFI loss (*p* < 0.01), and 16%, 43%, 61%, and 82%, respectively, for SFI loss (*p* < 0.01). Therefore, VFI and SFI decreased with the severity of body weight loss. However, similar changes were not observed in PMI.

**TABLE 1:** Clinical and treatment characteristics.

Variables		*N* (%)
Patient factors		
Age^a^	Years	64 (56–71)
Sex		
	Female	124 (36.1)
	Male	219 (63.8)
Pre‐CRT body weight (kg)^a^		60.4 (53.1–67.5)
Post‐CRT body weight (kg)^a^		59.0 (50.8–66.0)
Magnitude of %body weight^a^		−1.9 (−5.7–0.9)
Pre‐CRT BMI (kg/m^2^)^a^		22.7 (20.8–24.8)
Post‐CRT BMI (kg/m^2^)^a^		22.0 (20.3–24.1)
Magnitude of %BMI change^a^		−1.9 (−5.7–0.9)
Pre‐CRT albumin (g/dL)^a^		4.1 (3.9–4.4)
Post‐CRT albumin (g/dL)^a^		3.8 (3.6–4.0)
Pre‐CRT PMI (cm^2^/m^2^)^a^		5.7 (4.8–6.9)
Post‐CRT PMI (cm^2^/m^2^)^a^		5.6 (4.6–6.8)
Magnitude of %PMI change^a^		−2.6 (−9.1–4.1)
Pre‐CRT sarcopenia		133 (38.8)
Post‐CRT sarcopenia		150 (43.8)
Pre‐CRT VFI (cm^2^/m^2^)^a^		25.4 (12.7–44.2)
Post‐CRT VFI (cm^2^/m^2^)^a^		21.7 (9.2–36.8)
Magnitude of %VFI change^a^		−11.9 (−29.2–6.3)
Pre‐CRT SFI (cm^2^/m^2^)^a^		32.5 (22.1–48.4)
Post‐CRT SFI (cm^2^/m^2^)^a^		30.2 (20.6–42.6)
Magnitude of %SFI change^a^		−7.3 (−22.0–6.6)
ASA‐PS score	I	147 (42.8)
	II	189 (55.1)
	III	7 (2.1)
Charlson comorbidity index	0	257 (74.9)
	1	55 (16.0)
	≥ 2	31 (9.1)
Perioperative therapy		
Chemoradiotherapy regimen	Tegafur/Uracil + Leucovorin	255 (74.3)
	Tegafur/Uracil + Leucovorin + CPT‐11	78 (22.7)
	Tegafur/Gimeracil/Oteracil	4 (1.2)
	Tegafur/Gimeracil/Oteracil + Oxaliplatin	6 (1.8)
Postoperative chemotherapy	Present	110 (32.1)
	Absent	233 (67.9)
Surgical factors		
Surgical approach	Open	107 (31.2)
	Laparoscopic	126 (36.7)
	Robot	110 (32.1)
Surgical procedure	Low anterior resection	208 (60.6)
	Abdominal perineal resection	79 (23.0)
	Intersphincteric resection	48 (14.0)
	Total pelvic exenteration	4 (1.2)
	Hartmann's procedure	4 (1.2)
Stoma creation	No	96 (28.0)
	Ileostomy	141 (41.1)
	Colostomy	106 (30.9)
Lateral pelvic node dissection	Present	63 (18.4)
Tumor factors		
Primary tumor location from AV (cm)^a^		4.0 (3.0–6.0)
cT‐stage	2	5 (1.5)
	3	301 (87.7)
	4	37 (10.8)
cN‐stage	0	172 (50.1)
	≥ 1	171 (49.9)
ypT‐Stage	Complete response	36 (10.5)
	Tis	9 (2.6)
	1	26 (7.6)
	2	98 (28.6)
	3	152 (44.3)
	4	22 (6.4)
ypN‐Stage	0	251 (73.2)
	≥ 1	92 (26.8)
Histopathological type	Differentiated (well/moderate)	319 (93.0)
	Others (por, sig, muc)	24 (7.0)
Pathological response to preoperative chemotherapy	2/3	188 (54.8)
	0/1a/1b	155 (45.2)
Pre‐CRT serum CEA (ng/mL)^a^		6.3 (3.3–13.6)
Post‐CRT serum CEA (ng/mL)^a^		3.6 (2.4–5.4)

*Note:* Values in parentheses are percentages, unless indicated otherwise.

Abbreviations: ASA‐PS, American society of Anesthesiologists physical status; AV, anal verge; BMI, body mass index; CEA, carcinoembryonic antigen; CRT, chemoradiotherapy.

^a^ Values are medians (interquartile ranges).

### 3.2 Relationship Between Body Weight Loss and Clinicopathological Characteristics

Regarding patient characteristics, the body weight loss ≥ 10% group had a significantly higher pre‐CRT BMI, larger decreases in %VFI and %SFI, and a more advanced clinical T‐stage (cT4). At post‐CRT, the body weight loss ≥ 10% group had a lower BMI and serum albumin levels, a higher sarcopenia ratio, a higher incidence of lymph node metastasis, and was more likely to have an advanced pathological T‐stage (ypT3 and 4) (Table 2).

**TABLE 2:** Relationships between perioperative characteristics and body weight loss.

Variables		Body weight loss < 10%	Body weight loss ≥ 10%	*p* value
		(*n* = 302)	(*n* = 41)	
Patient factors				
Age^a^	Years	64 (56–71)	65 (57–73)	0.755
Sex	Female	105 (34.8)	19 (46.3)	0.154
	Male	197 (65.2)	22 (53.7)	
Pre‐CRT BMI (kg/m^2^)^a^		22.5 (20.7–24.7)	23.4 (21.5–26.0)	0.038
Magnitude of %BMI change^a^		−1.2 (−4.1–1.5)	−12.6 (−13.7 to −11.2)	< 0.001
Magnitude of %PMI change^a^		−2.8 (−9.0–4.3)	−0.94 (−10.0–3.3)	0.977
Magnitude of %VFI change^a^		−9.6 (−27.1–8.2)	−30.7 (−46.6 to −12.8)	< 0.001
Magnitude of %SFI change^a^		−5.3 (−17.6–8.4)	−27.2 (−41.5 to −17.3)	< 0.001
Pre‐CRT sarcopenia	Present	113 (37.4)	20 (48.8)	0.166
	Absent	189 (62.6)	21 (51.2)	
Post‐CRT sarcopenia	Present	126 (41.7)	24 (58.5)	0.043
	Absent	176 (58.3)	17 (41.5)	
Pre‐CRT albumin (g/dL)^a^		4.1 (3.9–4.4)	4.2 (3.9–4.4)	0.515
Post‐CRT albumin (g/dL)^a^		3.8 (3.6–4.1)	3.7 (3.3–4.0)	0.012
ASA‐PS score	I	132 (43.7)	15 (36.6)	0.384
	≥ II	170 (56.3)	26 (63.4)	
Charlson comorbidity index	0/1	276 (91.4)	36 (87.8)	0.470
	≥ 2	26 (8.6)	5 (12.2)	
Perioperative therapy				
CRT regimen	Tegafur/Uracil + Leucovorin	222 (73.5)	33 (80.5)	0.392
	Tegafur/Uracil + Leucovorin + CPT‐11	70 (23.2)	8 (19.5)	
	Tegafur/Gimeracil/Oteracil	4 (1.3)	0	
	Tegafur/Gimeracil/Oteracil + Oxaliplatin	6 (2.0)	0	
Adjuvant chemotherapy	Present	94 (31.1)	16 (39.0)	0.316
	Absent	208 (68.9)	25 (61.0)	
Surgical factors				
Surgical approach	Open	92 (30.5)	15 (36.6)	0.433
	Laparoscopic/Robotic	210 (69.5)	26 (63.4)	
Surgical procedure	Low anterior resection	188 (62.2)	20 (48.8)	0.137
	Abdominal perineal resection	70 (23.2)	9 (22.0)	
	Intersphincteric resection	39 (12.9)	9 (22.0)	
	Total pelvic exenteration	2 (0.7)	2 (4.8)	
	Hartmann's procedure	3 (1.0)	1 (2.4)	
Lateral pelvic node dissection	Present	52 (17.2)	11 (26.8)	0.153
	Absent	250 (82.8)	30 (73.2)	
Tumor factors				
Primary tumor location from AV (cm)^a^		4.0 (2.9–6.0)	5.0 (3.0–5.0)	0.723
cT‐stage	2/3	274 (90.7)	32 (78.0)	0.026
	4	28 (9.3)	9 (22.0)	
cN‐stage	0	156 (51.7)	16 (39.0)	0.128
	≥ 1	146 (48.3)	25 (61.0)	
ypT‐Stage	Complete response	33 (10.9)	3 (7.3)	0.203
	Tis	8 (2.7)	1 (2.5)	
	1	23 (7.6)	3 (7.3)	
	2	91 (30.1)	7 (17.1)	
	3	131 (43.4)	21 (51.2)	
	4	16 (5.3)	6 (14.6)	
ypN‐Stage	0	229 (75.8)	22 (53.7)	0.004
	≥ 1	73 (24.2)	19 (46.3)	
Histopathological type	Differentiated (well/moderate)	282 (93.4)	37 (90.2)	0.481
	Others (por, sig, muc)	20 (6.6)	4 (9.8)	
Pathological response to CRT	2/3	168 (55.6)	20 (48.8)	0.409
	0/1a/1b	134 (44.4)	21 (51.2)	
Pre‐CRT serum CEA (ng/mL)^a^		6.2 (3.3–13.4)	7.3 (3.4–14.4)	0.415
Post‐CRT serum CEA (ng/mL)^a^		3.6 (2.4–5.5)	3.6 (2.6–5.6)	0.676

*Note:* Values in parentheses are percentages, unless indicated otherwise.

Abbreviations: ASA‐PS, American society of Anesthesiologists physical status; AV, anal verge; BMI, body mass index; CEA, carcinoembryonic antigen; CRT, chemoradiotherapy; PMI, psoas muscle mass index; SFI, subcutaneous fat index; VFI, visceral fat index.

^a^ Values are medians (interquartile ranges).

### 3.3 Relationship Between Body Weight Loss and Adverse Events During CRT

We then evaluated the relationship between body weight loss and adverse events during CRT (Table S1). Severe adverse events (≥ grade 3) were significantly observed in patients with body weight loss ≥ 10% than those with body weight loss < 10%, especially neutropenia ≥ grade 3 was observed commonly. In addition, leukopenia ≥ grade 3 and diarrhea ≥ grade 3 tended to be more observed in patients with body weight loss ≥ 10% during CRT.

### 3.4 Effects of Changes in Body Comparison on Prognosis

**FIGURE 3:**
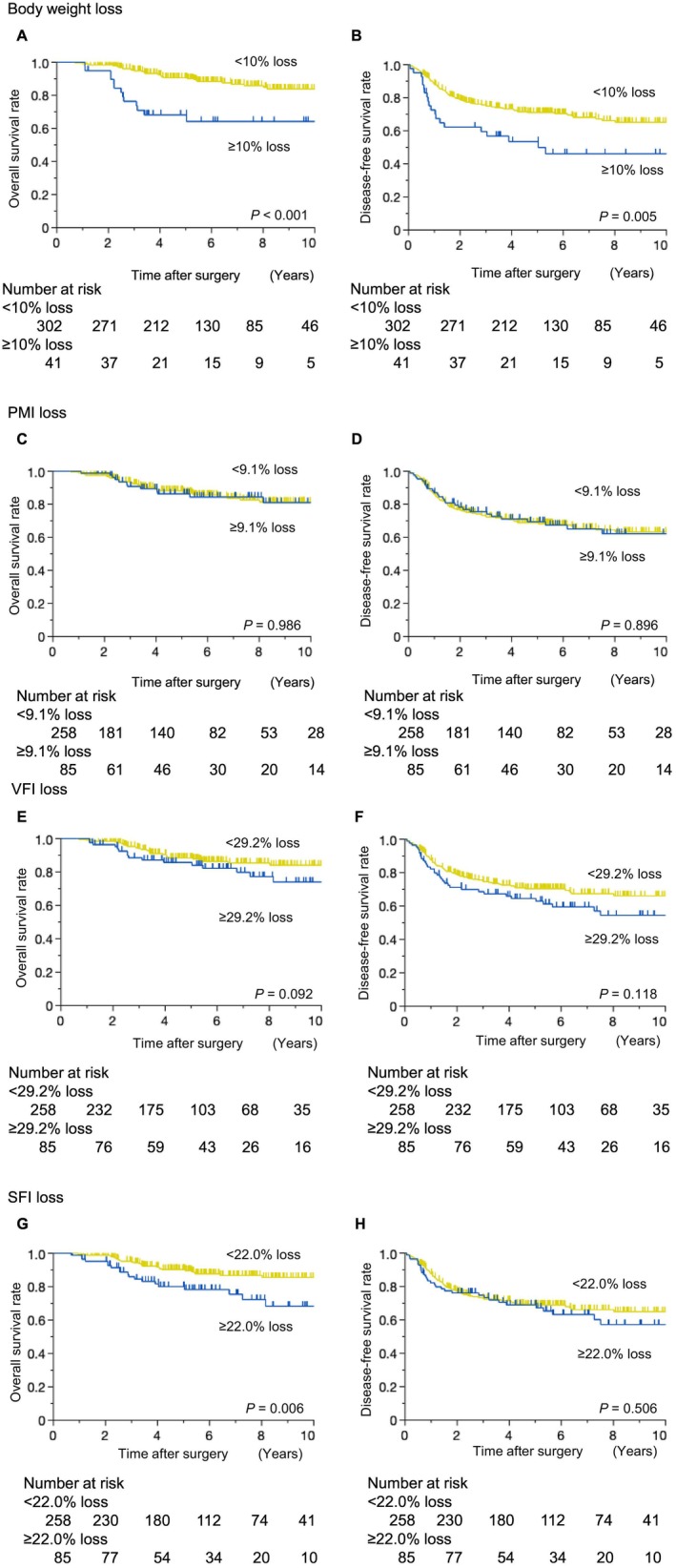
Survival outcomes according to changes in body composition. (A) Overall survival and (B) disease‐free survival according to body weight loss. (C) Overall survival and (D) disease‐free survival according to PMI loss. (E) Overall survival and (F) disease‐free survival according to VFI loss. (G) Overall survival and (H) disease‐free survival according to SFI loss.

Patients with body weight loss ≥ 10% had significantly lower 5‐year OS (68.2% vs. 90.5%; *p* < 0.001; Figure 3A) and 5‐year DFS (53.5% vs. 70.9%; *p* = 0.005; Figure 3B) rates than those with body weight loss < 10%. Similar results were obtained in a sensitivity analysis based on the treatment era; patients with body weight loss ≥ 10% had poor long‐term results (Figure S3). In addition, patients with body weight loss ≥ 10% had significantly shorter CSS (*p* < 0.001; Figure S4A) and RFS (*p* < 0.001; Figure S4B) than those with body weight loss < 10%. The long‐term prognosis of patients, namely, OS and DFS, generally worsened in relation to the degree of weight loss (Figure S5). Conversely, no significant differences were observed in OS or DFS rates between patients with PMI loss ≥ 9.1% and those with PMI loss < 9.1% (Figure 3C,D). The 5‐year OS rate was lower in patients with VFI loss ≥ 29.2% than in those with VFI loss < 29.2% (85.7% vs. 88.3%; *p* = 0.092; Figure 3E). The 5‐year DFS rate was lower in patients with visceral fat loss ≥ 29.2% than in those with visceral fat loss < 29.2% (64.5% vs. 70.4%; *p* = 0.1118; Figure 3F). Regarding SFI, patients with SFI loss ≥ 22.0% had a significantly lower 5‐year OS rate than those with SFI loss < 22.0% (74.3% vs. 88.3%; *p* = 0.006; Figure 3G). No significant difference was observed in the 5‐year DFS rate between patients with SFI loss ≥ 22.0% and those with SFI loss < 22.0% (69.0% vs. 68.8.8%; *p* = 0.506; Figure 3H).

Multivariate analyses revealed body weight loss ≥ 10% (hazard ratio [HR] 2.98; 95% CI 1.53–5.80; *p* = 0.001) and SFI loss ≥ 22.0% (HR 2.84; 95% CI 1.54–5.19; *p* = 0.007) as independent predictors of worse OS, along with a lower tumor location, an advanced tumor depth, the presence of node metastasis, and histopathological types other than differentiated (Table 3). Additionally, multivariate analyses showed that body weight loss ≥ 10% was a significant predictor of worse DFS (HR 1.79; 95% CI 1.13–2.86, *p* = 0.014, Table 4) along with a lower tumor location, an advanced tumor depth, the presence of node metastasis, a poor pathological response to CRT, and high serum CEA post‐CRT.

**TABLE 3:** Univariate and multivariate analyses of predictors of overall survival.

	Univariate analysis			Multivariate analysis (model 1)			Multivariate analysis (model 2)		
	HR	95% CI	*p* value	HR	95% CI	*p* value	HR	95% CI	*p* value
Age (years)									
≥ 65 vs. < 65	1.47	0.83–2.63	0.189						
Sex									
Male vs. female	1.25	0.68–2.31	0.475						
Charlson comorbidity index									
≥ 1 vs. 0	1.09	0.56–2.10	0.804						
Post‐CRT BMI (kg/m^2^)									
< 18.5 vs. ≥ 18.5	1.00	0.39–2.52	0.996						
Post‐CRT sarcopenia									
Present vs. absent	1.69	0.95–3.00	0.075						
Postoperative complications (Clavien‐Dindo classification)									
≥ Grade 3 vs. Grade 0/1/2	1.07	0.43–2.68	0.883						
Distance from AV (cm)									
< 5.0 vs. ≥ 5.0	2.04	1.10–3.77	0.023	2.20	1.18–4.10	0.013	2.28	1.23–4.24	0.009
ypT‐stage									
ypT4 vs. CR‐ypT3	4.20	1.96–9.00	< 0.001	2.73	1.17–6.38	0.020	3.35	1.49–7.74	0.005
ypN‐stage									
≥ 1 vs. 0	3.71	2.09–6.60	< 0.001	3.29	1.82–5.95	< 0.001	3.50	1.94–6.30	< 0.001
Histopathological type									
Others (por, sig, muc) vs. differentiated (well‐ and moderate)	5.24	2.66–10.3	< 0.001	5.14	1.54–10.4	< 0.001	5.80	2.89–11.7	< 0.001
Pathological response to CRT									
1a/1b/0 vs. 3/2	2.09	1.15–3.80	0.015	1.42	0.75–2.68	0.278	1.63	0.86–3.08	0.132
Adjuvant chemotherapy									
Present vs. absent	1.69	0.92–3.12	0.091						
Post‐CRT serum CEA (ng/mL)									
≥ 5.0 vs. < 5.0	1.25	0.68–2.31	0.472						
Body weight loss during CRT									
≥ 10% vs. < 10%	3.39	1.88–6.43	< 0.001	2.98	1.53–5.80	0.001			
PMI loss during CRT									
≥ 9.1% vs. < 9.1%	0.99	0.51–1.92	0.986						
VFI loss during CRT									
≥ 29.2% vs. < 29.2%	1.66	0.92–3.01	0.095						
SFI loss during CRT									
≥ 22.0% vs. < 22.0%	2.23	1.24–3.99	0.007				2.84	1.54–5.19	0.007

Abbreviations: AV, anal verge; BMI, body mass index; CEA, carcinoembryonic antigen; CI, confidential interval; CRT, chemoradiotherapy; HR, hazard ratio; PMI, psoas muscle mass index; SFI, subcutaneous fat index; VFI, visceral fat index.

**TABLE 4:** Univariate and multivariate analyses of predictors of disease‐free survival.

	Univariate analysis			Multivariate analysis		
	HR	95% CI	*p* value	HR	95% CI	*p* value
Age (years)						
≥ 65 vs. < 65	1.42	0.97–2.07	0.070			
Sex						
Male vs. female	1.16	0.78–1.73	0.453			
Charlson comorbidity index						
≥ 1 vs. 0	1.04	0.68–1.60	0.855			
Post‐CRT BMI (kg/m^2^)						
< 18.5 vs. ≥ 18.5	0.84	0.47–1.50	0.548			
Post‐CRT sarcopenia						
Present vs. absent	1.20	0.82–1.75	0.346			
Postoperative complications (Clavien‐Dindo classification)						
≥ Grade 3 vs. Grade 0/1/2	1.11	0.60–2.05	0.7495			
Distance from AV (cm)						
< 5.0 vs. ≥ 5.0	1.69	1.15–2.49	0.007	1.71	1.19–2.43	0.003
ypT‐stage						
ypT4 vs. CR‐ypT3	4.14	2.42–7.09	< 0.001	2.68	1.59–4.54	< 0.001
ypN‐stage						
≥ 1 vs. 0	2.63	1.80–3.86	< 0.001	2.01	1.38–2.92	< 0.001
Histopathological type						
Others (por, sig, muc) vs. differentiated (well‐ and moderate)	1.98	1.09–3.62	0.025	1.61	0.91–2.86	0.104
Pathological response to CRT						
0/1a/1b vs. 3/2	2.04	1.39–2.99	< 0.001	1.60	1.11–2.31	0.011
Adjuvant chemotherapy						
Present vs. absent	1.74	1.17–2.57	0.006	1.08	0.73–1.60	0.689
Post‐CRT serum CEA (ng/mL)						
≥ 5.0 vs. < 5.0	1.57	1.06–2.32	0.025	1.50	1.04–2.17	0.030
Body weight loss during CRT						
≥ 10% vs. < 10%	1.99	1.22–3.23	0.006	1.79	1.13–2.86	0.014
PMI loss during CRT						
≥ 9.1% vs. < 9.1%	1.03	0.67–1.58	0.896			
VFI loss during CRT						
≥ 29.2% vs. < 29.2%	1.38	0.92–2.07	0.119			
SFI loss during CRT						
≥ 22.0% vs. < 22.0%	1.15	0.76–1.76	0.507			

Abbreviations: AV, anal verge; BMI, body mass index; CEA, carcinoembryonic antigen; CI, confidential interval; CRT, chemoradiotherapy; HR, hazard ratio; PMI, psoas muscle mass index; SFI, subcutaneous fat index; VFI, visceral fat index.

### 3.5 Relationships Between Clinical Factors at Pre‐CRT for Body Weight Loss ≥ 10% During CRT

We investigated predictors of body weight loss ≥ 10% during CRT. Age and sex were adjusted for the evaluation of predictors because they have an impact on adverse events and physical conditions. Age‐ and sex‐adjusted logistic regression model analyses showed that pre‐CRT BMI ≥ 25 kg/m^2^ (OR 2.05; 95% CI 1.00–4.20, *p* = 0.04), pre‐CRT sarcopenia (OR 2.04; 95% CI 0.98–4.19, *p* = 0.051), and cT4 stage (OR 2.91; 95% CI 1.25–6.77, *p* = 0.01) had high OR for body weight loss ≥ 10% during CRT (Table 5).

**TABLE 5:** Univariate and multivariate analyses of predictors at pre‐CRT of body weight loss ≥ 10%.

	Univariate analysis			Age‐ and sex‐adjusted		
	OR	95% CI	*p* value	OR	95% CI	*p* value
Age (years)	1.16	0.23–5.89	0.856			
Sex						
Female vs. male	1.62	0.84–3.13	0.154			
Smoking						
Former/Current vs. never	0.73	0.38–1.41	0.354	0.89	0.42–1.85	0.748
Alcohol consumption						
Present vs. absent	0.53	0.21–1.30	0.139	0.52	0.21–1.30	0.159
Charlson comorbidity index						
≥ 2 vs. < 2	1.47	0.53–4.08	0.470	1.61	0.57–4.53	0.367
Pre‐CRT BMI (kg/m^2^)						
≥ 25.0 vs. < 25.0	1.93	0.96–3.89	0.075	2.05	1.00–4.20	0.049
Pre‐CRT sarcopenia						
Present vs. absent	1.59	0.83–3.07	0.164	2.04	0.998–4.19	0.051
Pre‐CRT albumin (g/dL)						
< 3.5 vs. ≥ 3.5	0.62	0.14–2.74	0.531	1.58	0.36–7.01	0.547
Distance from AV (cm)						
< 5.0 vs. ≥ 5.0	0.93	0.48–1.78	0.821	0.91	0.47–1.76	0.784
cT‐stage						
T4 vs. ≤ 3	2.75	1.19–6.35	0.017	2.91	1.25–6.77	0.013
cN‐stage						
≥ 1 vs. 0	1.67	0.86–3.25	0.132	1.64	0.84–3.22	0.147
Pre‐CRT serum CEA (ng/mL)						
≥ 5.0 vs. < 5.0	1.68	0.83–3.43	0.151	1.63	0.80–3.34	0.180

Abbreviations: AV, anal verge; BMI, body mass index; CEA, carcinoembryonic antigen; CI, confidential interval; CRT, chemoradiotherapy; OR, odds ratio.

## 4 Discussion

The present study demonstrated that body weight and SFI loss during CRT correlated with poor OS in patients with LARC undergoing radical surgery following CRT. Therefore, body composition changes during CRT are important yet distinct predictors. Additionally, body weight loss ≥ 10% during CRT was an independent predictor of worse DFS. Body weight loss during CRT may provide information in addition to well‐established prognostic factors, including the TNM stage and pathological response to CRT. Furthermore, body weight loss ≥ 10% may have a potential relationship with high pre‐CRT BMI, the pre‐CRT sarcopenia status, or cT4. Therefore, clinicians need to consider not only adverse events, but also patients' conditions and the nutritional intake status. To the best of our knowledge, this is the first study to examine the relationship between body weight and changes in body composition during CRT and poor long‐term outcomes and predictive factors for body weight loss in patients with LARC undergoing radical surgery following CRT.

Body weight loss and sarcopenia are considered typical findings as cancer cachexia [14]. Therefore, body weight loss, rather than BMI, has garnered more attention as a surrogate for the nutritional status in various cancers [15]. Although previous studies focused on BMI, Lin et al. [2] reported that BMI loss ≥ 7% during CRT was a prognostic marker for OS, which is consistent with the present results. Additionally, body weight loss during CRT has been identified as a prognostic marker in several cancers [16].

To elucidate changes in body composition during CRT in detail, we measured PMI, VFI, and SFI. The median loss in PMI was small (2.6), while those in VFI and SFI were larger (11.9 and 7.3, respectively). Therefore, body composition changes during CRT predominantly occurred in the fat compartment, which is consistent with previous findings [17]. In addition, further reductions were observed in VFI and SFI, but not in PMI, as body weight continued to decrease (Figure 2). These results suggest that body composition changes during CRT are complex; however, body weight loss has potential as a predictor of reductions in adipose tissue volume during CRT.

Body weight loss ≥ 10% during CRT was observed in patients with high BMI, a high clinical depth of tumors, and pretreatment sarcopenia pre‐CRT (Table 5). High levels of inflammatory cytokines correlate with invasion depth and proinflammatory cytokines have also been implicated in radiation‐induced gastrointestinal mucositis [18]. Bulky tumor‐treated radiotherapy has the potential to induce high levels of cytokines, such as IL‐6, which is related to the development of diarrhea in ovarian cancer patients [19]. This finding supports the present result showing that diarrhea was slightly more common in patients with body weight loss ≥ 10% during CRT (Table S1). On the other hand, in esophageal cancer, treatment‐related toxicities, such as mucositis, esophagitis, and anorexia, have been reported to induce body weight loss [20]. In rectal cancer, we previously reported that systemic inflammatory response markers increased during CRT [21]. These findings suggest that the mechanisms underlying body weight loss differ in various cancers; however, therapy‐induced cytokines or inflammation may contribute to body weight loss. A relationship has also been reported between inflammatory cytokines and skeletal muscle loss [22]. Sarcopenia has been linked to treatment toxicity in several malignancies [23]. Collectively, these findings imply that CRT‐induced cytokines or inflammation leads to severe body weight loss in patients with sarcopenia or advanced cancer and, thus, further large‐scale studies are warranted.

Although all parameters of body composition during CRT significantly decreased (Figure 1), the present results showed that PMI loss during CRT did not significantly impact long‐term outcomes. This result is consistent with the study by Liu et al. [24], but not with those by Chung et al. [6] and Levolger et al. [7]. There are several possible explanations for this discrepancy. Some patients experienced a small gain of skeletal muscle during CRT; however, this was not sufficient to improve survival. Furthermore, the timing of CT scans after CRT differed. In the present study, the interval between the completion of CRT and CT imaging was 3–5 weeks, whereas it was 4–12 weeks in the other studies. This interval may affect various factors, including skeletal muscle and fat mass. In addition, the method used to measure skeletal muscle differed. Therefore, the optimal method for measuring muscle mass requires further investigation.

Current evidence suggests that the mechanisms underlying the potential effects of visceral and subcutaneous fat in patients with cancer may differ. Visceral fat is associated with endocrine activity, promoting systemic inflammation and protein wasting, which leads to carcinogenesis and cancer progression [25, 26, 27]. Therefore, patients with low VFI have reported having better oncologic outcomes than those with high VFI [28]. While this was inconsistent with the results obtained herein, the findings, such as VFI loss during CRT related to worse OS reported by Kim et al. [5], support the present study. Therefore, the mechanisms underlying the effects of visceral fat loss during CRT on long‐term outcomes require further investigation. Conversely, subcutaneous fat predominantly produces leptin [29], whose loss increases insulin resistance associated with hyperinsulinemia and elevated C‐peptide, leading to a worse prognosis in breast [30] and prostate [31] cancers. Recent studies demonstrated that patients with low SFI had shorter OS than those with high SFI in various cancers [32]. In addition, Given the gluteal subcutaneous fat increase during CRT related to favorable clinical outcomes [33], SFI loss during CRT may result in poor outcomes among patients with rectal cancer who undergo CRT.

Beyond body weight and SFI loss, our analysis also identified several other predictive factors for OS, including post‐CRT TNM stage, histopathological type, and tumor location. Although the prognostic significance of these particular factors is not yet universally confirmed, our findings are consistent with previous reports. Frambach et al. identified ypT stage ≥ 2, positive lymph node status, and tumor location ≤ 5 cm from the anal verge as risk factors for recurrences following rectal resection after CRT [34]. Similarly, Liu et al. reported that pathological findings, including ypT stage ≥ 3, ypN ≥ 1, poor differentiation, and location less than 5 cm from the anal verge, are independent predictors of OS [32]. Poor or mucinous type and T4 stage tumors have been reported to be unresponsive to neoadjuvant CRT [35]. Consequently, an advanced T‐ or N‐stage after CRT is often considered to reflect a poor response to treatment, which may be attributed to more aggressive tumor biology and, thus, predispose patients to poor survival. Anatomical considerations may further elucidate the relationship between tumor location and the risk of distant relapse. Specifically, the dual venous drainage system of the rectum—where the superior rectal vein drains into the inferior mesenteric vein (connecting to the portal vein) and the inferior rectal vein drains into the internal iliac vein (connecting to the systemic circulation)—may influence the pathway and frequency of distant metastasis. However, to fully clarify the clinical implications and robustly confirm the prognostic value of these factors, further prospective studies are warranted.

This study has several potential limitations. This was a retrospective cohort study performed at a single institution. Furthermore, changes in actual muscle and fat measurements were not directly investigated. The definition of sarcopenia is based solely on muscle mass and does not follow the current diagnostic criteria of international working groups, such as EWGSOP2 (2018) or AWGS 2019, which include a muscle strength assessment. Moreover, the optimal interval between pre‐ and post‐CRT CT images for measuring skeletal muscle and adipose tissue has not yet been established.

## 5 Conclusion

Body weight loss ≥ 10% during CRT is a predictor of worse long‐term outcomes in patients with LARC who undergo radical surgery following CRT. Furthermore, pre‐CRT BMI ≥ 25 kg/m^2^ and cT4 were associated with higher odds of body weight loss ≥ 10% during CRT after adjustments for age and sex.

## Author Contributions

**Shinya Abe:** conceptualization, methodology, data curation, investigation, validation, formal analysis, writing – original draft, writing – review and editing. **Hiroaki Nozawa:** supervision, writing – review and editing, funding acquisition, methodology. **Kazuhito Sasaki:** methodology, supervision, writing – review and editing. **Koji Murono:** supervision, writing – review and editing. **Shigenobu Emoto:** supervision, writing – review and editing. **Yuichiro Yokoyama:** data curation, writing – review and editing, supervision. **Yuzo Nagai:** supervision, data curation, writing – review and editing. **Yuzo Harada:** supervision, writing – review and editing. **Takahide Shinagawa:** supervision, writing – review and editing. **Soichiro Ishihara:** conceptualization, methodology, funding acquisition, writing – review and editing, supervision.

## Funding

This research was supported by Grants‐in‐Aid for Scientific Research (C: Grant 24K10447, 24K10422, 24K10376, 23K06607, and 22K08793) from the Japan Society for the Promotion of Science.

## Disclosure

The authors have nothing to report.

## Ethics Statement

The present study was approved by the Institutional Ethics Committee of The University of Tokyo (No. 3252‐[15]).

## Consent

Informed consent was obtained through an opt‐out method.

## Conflicts of Interest

The authors declare no conflicts of interest.

## Supporting information


**Figure S1:** Study cohort selection process. Three hundred and 78 patients with clinical stage II–IV rectal cancer treated with preoperative chemotherapy were enrolled between April 2005 and March 2023. Three hundred and 43 patients were included in the final study population.
**Figure S2:** The percentages of cases under the median of changes in body composition according to body weight loss (WL). (A) The psoas muscle mass index, (B) visceral fat index, and (C) subcutaneous fat index.
**Figure S3:** Survival outcomes according to changes in body weight loss in each treatment era. Overall survival between 2005 and 2011 years (A), 2012 and 2017 years (B), and 2018 and 2023 years (C). Disease‐free survival between 2005 and 2011 years (D), 2012 and 2017 years (E), and 2018 and 2023 years (F).
**Figure S4:** Survival outcomes according to body weight loss. (A) Cancer‐specific survival and (B) recurrence‐free survival.
**Figure S5:** Relationship between body weight loss during chemoradiotherapy and overall survival (OS) and disease‐free survival (DFS). Kaplan–Meier (A) OS and (B) DFS curves stratified according to the degree of body weight loss. To address the issue of multiple comparisons, the Holm method was applied.

**Table S1:** Relationships between body weight loss and adverse events during CRT.

## References

[1] H. A. Wolff , L. C. Conradi , T. Beissbarth , et al., “Gender Affects Acute Organ Toxicity During Radiochemotherapy for Rectal Cancer: Long‐Term Results of the German CAO/ARO/AIO‐94 Phase III Trial,” Radiotherapy and Oncology 108, no. 1 (2013): 48–54, 10.1016/j.radonc.2013.05.009. 23768685

[2] J. Lin , J. Peng , A. Qdaisat , et al., “Severe Weight Loss During Preoperative Chemoradiotherapy Compromises Survival Outcome for Patients With Locally Advanced Rectal Cancer,” Journal of Cancer Research and Clinical Oncology 142, no. 12 (2016): 2551–2560, 10.1007/s00432-016-2225-1. 27613188 PMC5095158

[3] C. M. Prado , J. R. Lieffers , L. J. McCargar , et al., “Prevalence and Clinical Implications of Sarcopenic Obesity in Patients With Solid Tumours of the Respiratory and Gastrointestinal Tracts: A Population‐Based Study,” Lancet Oncology 9, no. 7 (2008): 629–635, 10.1016/S1470-2045(08)70153-0. 18539529

[4] X. Y. Xu , X. M. Jiang , Q. Xu , et al., “Skeletal Muscle Change During Neoadjuvant Therapy and Its Impact on Prognosis in Patients With Gastrointestinal Cancers: A Systematic Review and Meta‐Analysis,” Frontiers in Oncology 12 (2022): 892935, 10.3389/fonc.2022.892935. 35692760 PMC9186070

[5] S. I. Kim , S. Yoon , T. M. Kim , J. Y. Cho , H. H. Chung , and Y. S. Song , “Prognostic Implications of Body Composition Change During Primary Treatment in Patients With Ovarian Cancer: A Retrospective Study Using an Artificial Intelligence‐Based Volumetric Technique,” Gynecologic Oncology 162, no. 1 (2021): 72–79, 10.1016/j.ygyno.2021.05.004. 33994146

[6] E. Chung , H. S. Lee , E. S. Cho , et al., “Prognostic Significance of Sarcopenia and Skeletal Muscle Mass Change During Preoperative Chemoradiotherapy in Locally Advanced Rectal Cancer,” Clinical Nutrition 39, no. 3 (2020): 820–828, 10.1016/j.clnu.2019.03.014. 30928250

[7] S. Levolger , M. G. van Vledder , W. J. Alberda , et al., “Muscle Wasting and Survival Following Pre‐Operative Chemoradiotherapy for Locally Advanced Rectal Carcinoma,” Clinical Nutrition 37, no. 5 (2018): 1728–1735, 10.1016/j.clnu.2017.06.028. 28756039

[8] K. Kawai , H. Shiratori , K. Hata , et al., “Optimal Size Criteria for Lateral Lymph Node Dissection After Neoadjuvant Chemoradiotherapy for Rectal Cancer,” Diseases of the Colon & Rectum 64, no. 3 (2021): 274–283, 10.1097/DCR.0000000000001866. 33395141

[9] Rectum JSfCotCa , “Japanese Classification of Colorectal, Appendiceal, and Anal Carcinoma: The 3d English Edition [Secondary Publication],” Journal of Anus, Rectum and Colon 3, no. 4 (2019): 175–195, 10.23922/jarc.2019-018. PMC6845287 31768468

[10] Y. Hashiguchi , K. Muro , Y. Saito , et al., “Japanese Society for Cancer of the Colon and Rectum (JSCCR) Guidelines 2019 for the Treatment of Colorectal Cancer,” International Journal of Clinical Oncology 25, no. 1 (2020): 1–42, 10.1007/s10147-019-01485-z. 31203527 PMC6946738

[11] Y. C. Choi , P. C. Chan , K. A. Cheung , et al., “Impact of Weight Loss on Treatment Interruption and Unplanned Hospital Admission in Head and Neck Cancer Patients Undergoing Curative (Chemo)‐Radiotherapy in Hong Kong,” Supportive Care in Cancer 31, no. 8 (2023): 487, 10.1007/s00520-023-07952-8. 37486576

[12] S. Abe , K. Kawai , H. Nozawa , et al., “Preoperative Sarcopenia Is a Poor Prognostic Factor in Lower Rectal Cancer Patients Undergoing Neoadjuvant Chemoradiotherapy: A Retrospective Study,” International Journal of Clinical Oncology 27, no. 1 (2022): 141–153, 10.1007/s10147-021-02062-z. 34741193

[13] Y. Hamaguchi , T. Kaido , S. Okumura , et al., “Proposal for New Diagnostic Criteria for Low Skeletal Muscle Mass Based on Computed Tomography Imaging in Asian Adults,” Nutrition 32, no. 11–12 (2016): 1200–1205, 10.1016/j.nut.2016.04.003. 27292773

[14] K. Fearon , F. Strasser , S. D. Anker , et al., “Definition and Classification of Cancer Cachexia: An International Consensus,” Lancet Oncology 12, no. 5 (2011): 489–495, 10.1016/S1470-2045(10)70218-7. 21296615

[15] J. Jou , E. Coulter , T. Roberts , et al., “Assessment of Malnutrition by Unintentional Weight Loss and Its Implications on Oncologic Outcomes in Patient With Locally Advanced Cervical Cancer Receiving Primary Chemoradiation,” Gynecologic Oncology 160, no. 3 (2021): 721–728, 10.1016/j.ygyno.2020.12.009. 33342621

[16] P. Naumann , J. Eberlein , B. Farnia , T. Hackert , J. Debus , and S. E. Combs , “Continued Weight Loss and Sarcopenia Predict Poor Outcomes in Locally Advanced Pancreatic Cancer Treated With Chemoradiation,” Cancers 11, no. 5 (2019): 709, 10.3390/cancers11050709. 31126040 PMC6562489

[17] M. Sandini , M. Patino , C. R. Ferrone , et al., “Association Between Changes in Body Composition and Neoadjuvant Treatment for Pancreatic Cancer,” JAMA Surgery 153, no. 9 (2018): 809–815, 10.1001/jamasurg.2018.0979. 29801062 PMC6583880

[18] D. K. Kim , S. Y. Oh , H. C. Kwon , et al., “Clinical Significances of Preoperative Serum Interleukin‐6 and C‐Reactive Protein Level in Operable Gastric Cancer,” BMC Cancer 9 (2009): 155, 10.1186/1471-2407-9-155. 19457231 PMC2694817

[19] K. Y. Eom , C. W. Wee , C. Song , et al., “The Association Between Diarrhea and Serum Cytokines in Patients With Gynecologic Cancer Treated With Surgery and Pelvic Chemoradiotherapy,” Clinical and Translational Radiation Oncology 29 (2021): 60–64, 10.1016/j.ctro.2021.05.010. 34159263 PMC8203500

[20] T. T. Huang , S. Y. Chou , Y. H. Lin , et al., “The Impact of Weight Loss During Chemoradiotherapy for Unresectable Esophageal Cancer: Real‐World Results,” Life 12, no. 5 (2022): 706, 10.3390/life12050706. 35629373 PMC9146706

[21] S. Abe , H. Nozawa , K. Kawai , et al., “Poor Nutrition and Sarcopenia Are Related to Systemic Inflammatory Response in Patients With Rectal Cancer Undergoing Preoperative Chemoradiotherapy,” International Journal of Colorectal Disease 37, no. 1 (2022): 189–200, 10.1007/s00384-021-04039-w. 34633498

[22] J. M. Argilés , S. Busquets , and F. J. López‐Soriano , “The Pivotal Role of Cytokines in Muscle Wasting During Cancer,” International Journal of Biochemistry & Cell Biology 37, no. 10 (2005): 2036–2046, 10.1016/j.biocel.2005.03.014. 16105746

[23] A. Surov , M. Pech , D. Gessner , et al., “Low Skeletal Muscle Mass Is a Predictor of Treatment Related Toxicity in Oncologic Patients. A Meta‐Analysis,” Clinical Nutrition 40, no. 10 (2021): 5298–5310, 10.1016/j.clnu.2021.08.023. 34536638

[24] Z. Liu , S. Lu , Y. Wang , et al., “Impact of Body Composition During Neoadjuvant Chemoradiotherapy on Complications, Survival and Tumor Response in Patients With Locally Advanced Rectal Cancer,” Frontiers in Nutrition 9 (2022): 796601, 10.3389/fnut.2022.796601. 35155538 PMC8830534

[25] J. M. Argilés , S. Busquets , B. Stemmler , and F. J. López‐Soriano , “Cachexia and Sarcopenia: Mechanisms and Potential Targets for Intervention,” Current Opinion in Pharmacology 22 (2015): 100–106, 10.1016/j.coph.2015.04.003. 25974750

[26] H. Tilg and A. R. Moschen , “Adipocytokines: Mediators Linking Adipose Tissue, Inflammation and Immunity,” Nature Reviews. Immunology 6, no. 10 (2006): 772–783, 10.1038/nri1937. 16998510

[27] E. E. Frezza , M. S. Wachtel , and M. Chiriva‐Internati , “Influence of Obesity on the Risk of Developing Colon Cancer,” Gut 55, no. 2 (2006): 285–291, 10.1136/gut.2005.073163. 16239255 PMC1856517

[28] L. Zuo , J. Lin , S. Ge , et al., “Preoperative Visceral Fat Index Predicts the Survival Outcomes of Patients With Gastric Cancer After Surgery,” Oncology Letters 27, no. 3 (2024): 99, 10.3892/ol.2024.14233. 38298425 PMC10829067

[29] Y. Cao , “Angiogenesis Modulates Adipogenesis and Obesity,” Journal of Clinical Investigation 117, no. 9 (2007): 2362–2368, 10.1172/JCI32239. 17786229 PMC1963348

[30] M. L. Irwin , C. Duggan , C. Y. Wang , et al., “Fasting C‐Peptide Levels and Death Resulting From All Causes and Breast Cancer: The Health, Eating, Activity, and Lifestyle Study,” Journal of Clinical Oncology 29, no. 1 (2011): 47–53, 10.1200/JCO.2010.28.4752. 21115859 PMC3055859

[31] J. Ma , H. Li , E. Giovannucci , et al., “Prediagnostic Body‐Mass Index, Plasma C‐Peptide Concentration, and Prostate Cancer‐Specific Mortality in Men With Prostate Cancer: A Long‐Term Survival Analysis,” Lancet Oncology 9, no. 11 (2008): 1039–1047, 10.1016/S1470-2045(08)70235-3. 18835745 PMC2651222

[32] S. Liu , F. He , Y. Guan , et al., “Pathologic‐Based Nomograms for Predicting Overall Survival and Disease‐Free Survival Among Patients With Locally Advanced Rectal Cancer,” Cancer Management and Research 13 (2021): 1777–1789, 10.2147/CMAR.S296593. 33654427 PMC7910108

[33] W. Huang , Z. Feng , M. Ma , et al., “Different Impacts of Adipose Tissue Dynamics on Prognosis in Patients With Resectable Locally Advanced Rectal Cancer Treated With and Without Neoadjuvant Treatment,” Frontiers in Oncology 14 (2024): 1421651, 10.3389/fonc.2024.1421651. 39148902 PMC11324464

[34] P. Frambach , S. Pucciarelli , A. Perin , et al., “Metastatic Pattern and New Primary Tumours After Neoadjuvant Therapy and Surgery in Rectal Cancer,” Colorectal Disease 20, no. 12 (2018): O326–O334, 10.1111/codi.14427. 30230157

[35] M. Li , Q. Xiao , N. Venkatachalam , et al., “Predicting Response to Neoadjuvant Chemoradiotherapy in Rectal Cancer: From Biomarkers to Tumor Models,” Therapeutic Advances in Medical Oncology 14 (2022): 17588359221077972, 10.1177/17588359221077972. 35222695 PMC8864271

