# Supplementary material for: Impact of Body Weight Loss During Preoperative Chemoradiotherapy on Prognosis of Patients With Lower Rectal Cancer
Source: Ann Gastroenterol Surg. 2025 Dec 15;10(3):722–38. doi: 10.1002/ags3.70146 (PMC13178290; doi:10.1002/ags3.70146)
Supplement: Supplementary file 1 — Figure S1: Study cohort selection process. Three hundred and 78 patients with clinical stage II–IV rectal cancer treated with preoperative chemotherapy were enrolled between April 2005 and March 2023. Three hundred and 43 patients were included in the final study population. Figure S2: The percentages of cases under the median of changes in body composition according to body weight loss (WL). (A) The psoas muscle mass index, (B) visceral fat index, and (C) subcutaneous fat index. Figure S3: Survival outcomes according to changes in body weight loss in each treatment era. Overall survival between 2005 and 2011 years (A), 2012 and 2017 years (B), and 2018 and 2023 years (C). Disease‐free survival between 2005 and 2011 years (D), 2012 and 2017 years (E), and 2018 and 2023 years (F). Figure S4: Survival outcomes according to body weight loss. (A) Cancer‐specific survival and (B) recurrence‐free survival. Figure S5: Relationship between body weight loss during chemoradiotherapy and overall survival (OS) and disease‐free survival (DFS). Kaplan–Meier (A) OS and (B) DFS curves stratified according to the degree of body weight loss. To address the issue of multiple comparisons, the Holm method was applied. [file AGS3-10-722-s002.pptx]

## Slide 1
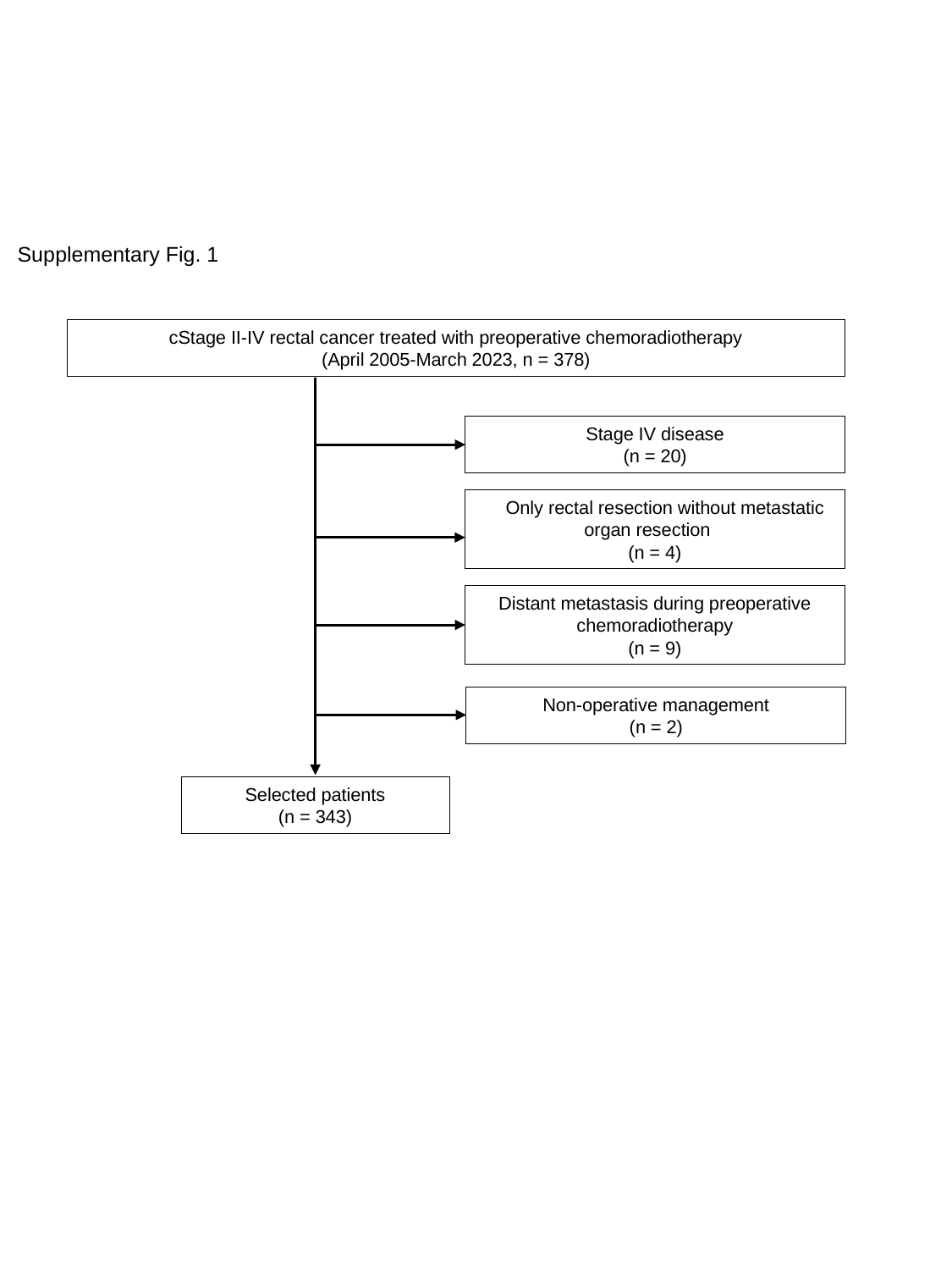

Supplementary Fig. 1
cStage II-IV rectal cancer treated with preoperative chemoradiotherapy
(April 2005-March 2023, n = 378)
Stage IV disease
(n = 20)
 Only rectal resection without metastatic organ resection
(n = 4)
Distant metastasis during preoperative chemoradiotherapy
(n = 9)
Non-operative management
(n = 2)
Selected patients
(n = 343)

## Slide 2
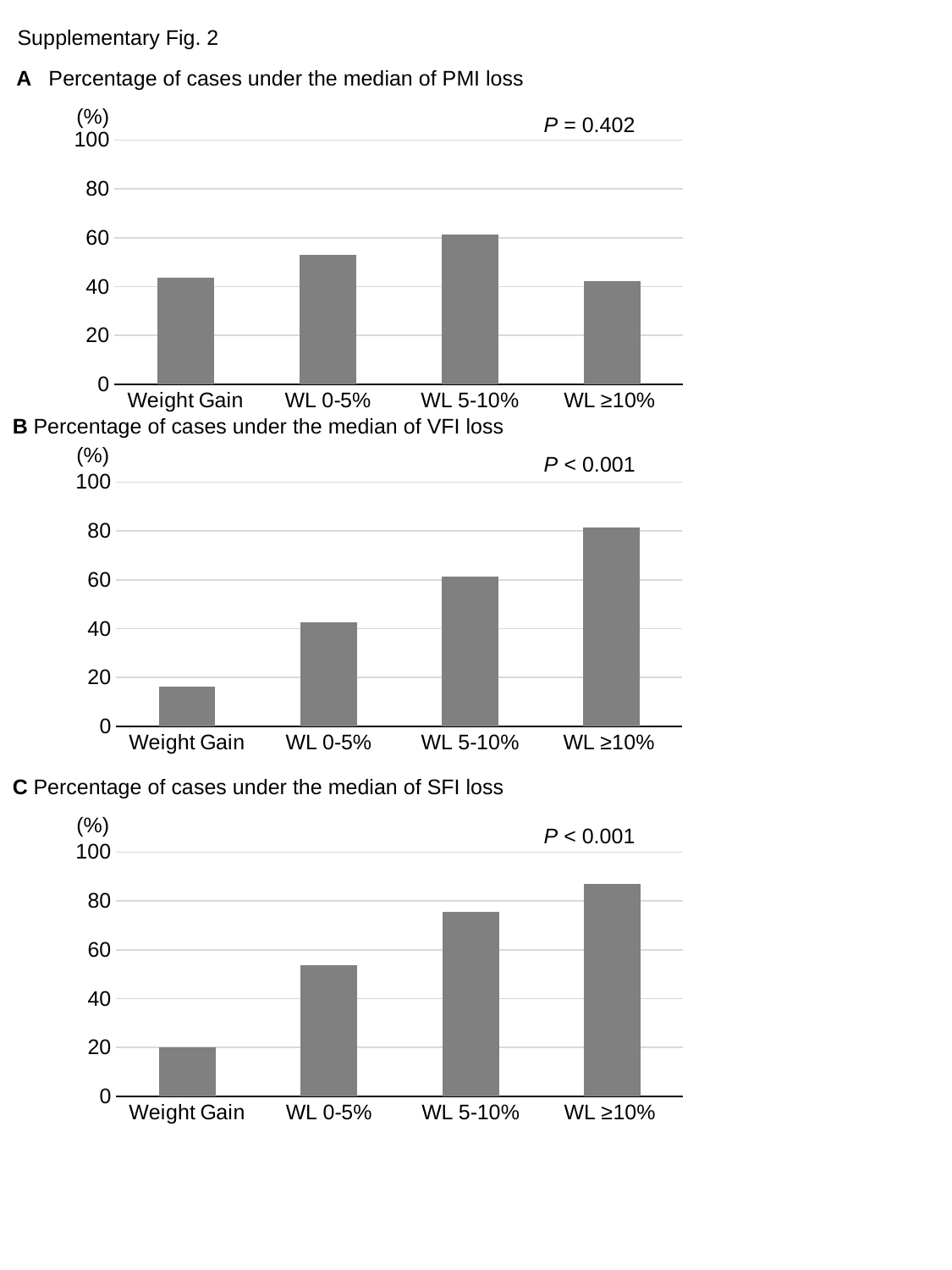

Supplementary Fig. 2
A Percentage of cases under the median of PMI loss
(%)
P = 0.402
### Chart
| Category | Below the median of PMI loss |
|---|---|
| Weight Gain | 43.63636363636363 |
| WL 0-5% | 52.89855072463768 |
| WL 5-10% | 61.40350877192983 |
| WL ≥10% | 42.10526315789473 |B Percentage of cases under the median of VFI loss
(%)
P < 0.001
### Chart
| Category | Below the median of VFI loss |
|---|---|
| Weight Gain | 16.363636363636363 |
| WL 0-5% | 42.7536231884058 |
| WL 5-10% | 61.40350877192983 |
| WL ≥10% | 81.57894736842105 |C Percentage of cases under the median of SFI loss
(%)
P < 0.001
### Chart
| Category | Below the median of SFI loss |
|---|---|
| Weight Gain | 20.0 |
| WL 0-5% | 53.62318840579711 |
| WL 5-10% | 75.43859649122807 |
| WL ≥10% | 86.8421052631579 |

## Slide 3
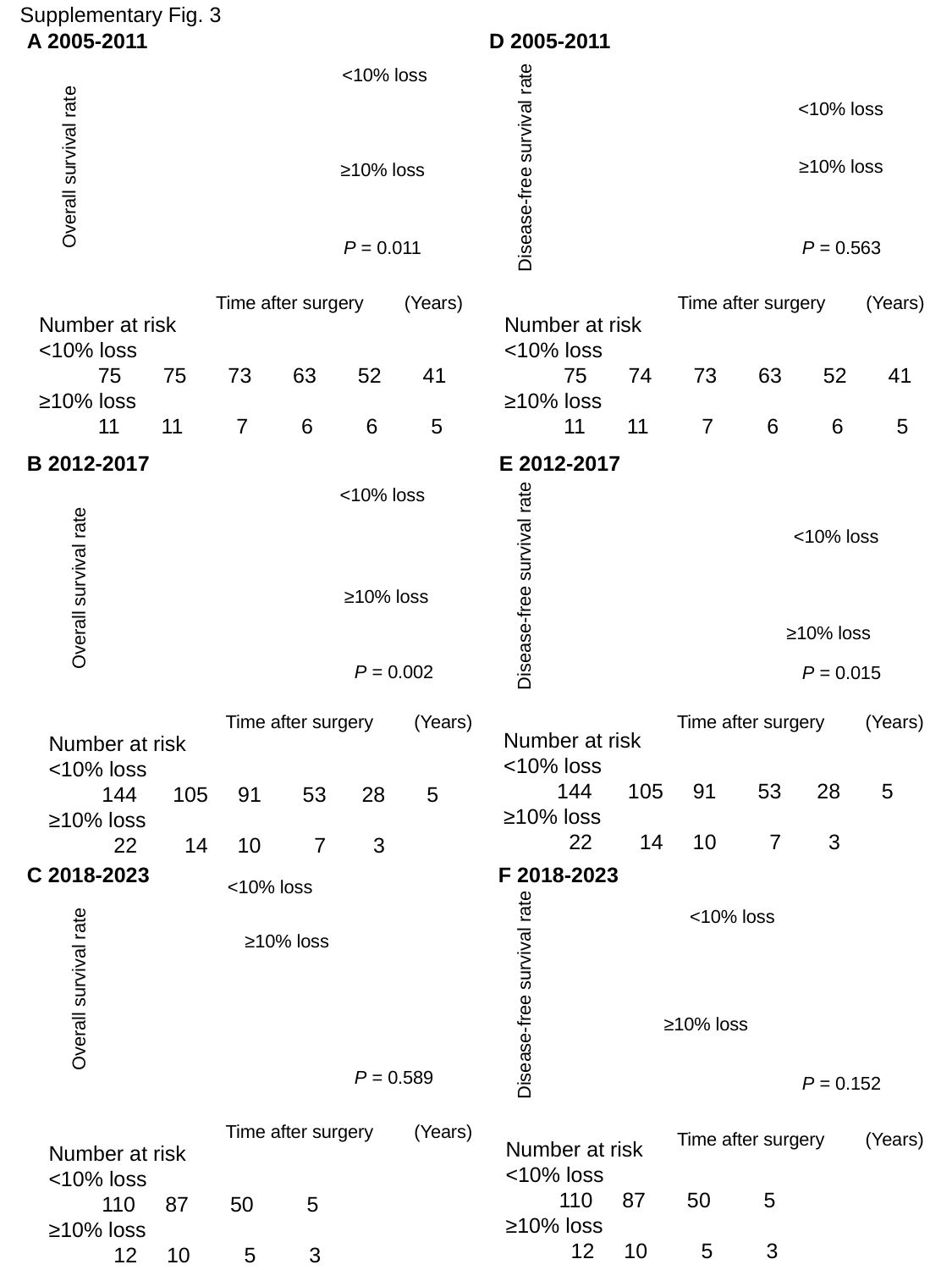

Supplementary Fig. 3
D 2005-2011
A 2005-2011
<10% loss
<10% loss
≥10% loss
Overall survival rate
Disease-free survival rate
≥10% loss
P = 0.011
P = 0.563
 Time after surgery (Years)
 Time after surgery (Years)
Number at risk
<10% loss
 75 74 73 63 52 41
≥10% loss
 11 11 7 6 6 5
Number at risk
<10% loss
 75 75 73 63 52 41
≥10% loss
 11 11 7 6 6 5
E 2012-2017
B 2012-2017
<10% loss
<10% loss
Disease-free survival rate
Overall survival rate
≥10% loss
≥10% loss
P = 0.002
P = 0.015
 Time after surgery (Years)
 Time after surgery (Years)
Number at risk
<10% loss
 144 105 91 53 28 5
≥10% loss
 22 14 10 7 3
Number at risk
<10% loss
 144 105 91 53 28 5
≥10% loss
 22 14 10 7 3
C 2018-2023
F 2018-2023
<10% loss
<10% loss
≥10% loss
Overall survival rate
Disease-free survival rate
≥10% loss
P = 0.589
P = 0.152
 Time after surgery (Years)
 Time after surgery (Years)
Number at risk
<10% loss
 110 87 50 5
≥10% loss
 112 10 55 13
Number at risk
<10% loss
 110 87 50 5
≥10% loss
 112 10 55 13

## Slide 4
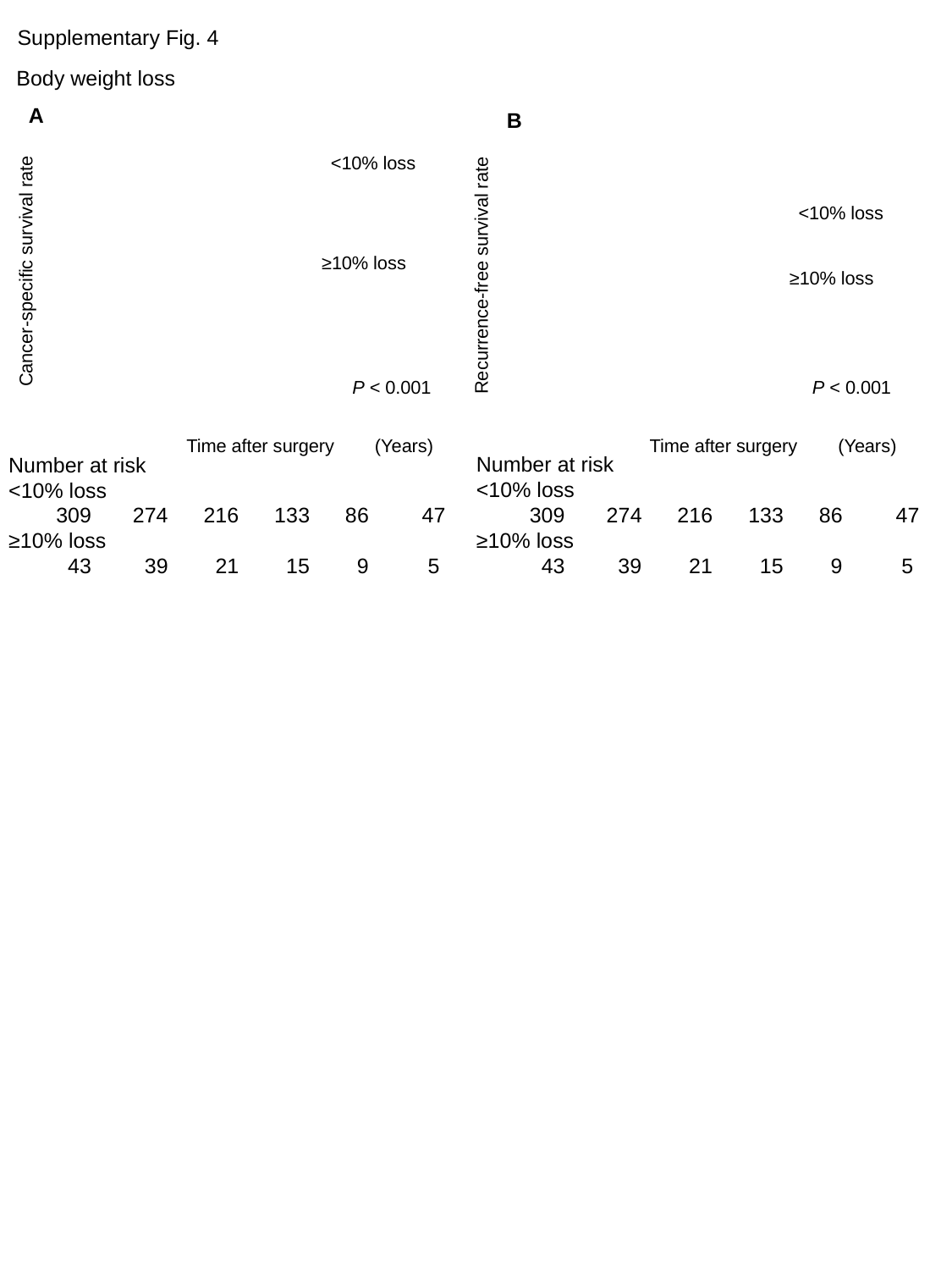

Supplementary Fig. 4
Body weight loss
A
B
<10% loss
<10% loss
≥10% loss
Cancer-specific survival rate
Recurrence-free survival rate
≥10% loss
P < 0.001
P < 0.001
 Time after surgery (Years)
 Time after surgery (Years)
Number at risk
<10% loss
 309 274 216 133 86 47
≥10% loss
 43 39 21 15 9 5
Number at risk
<10% loss
 309 274 216 133 86 47
≥10% loss
 43 39 21 15 9 5

## Slide 5
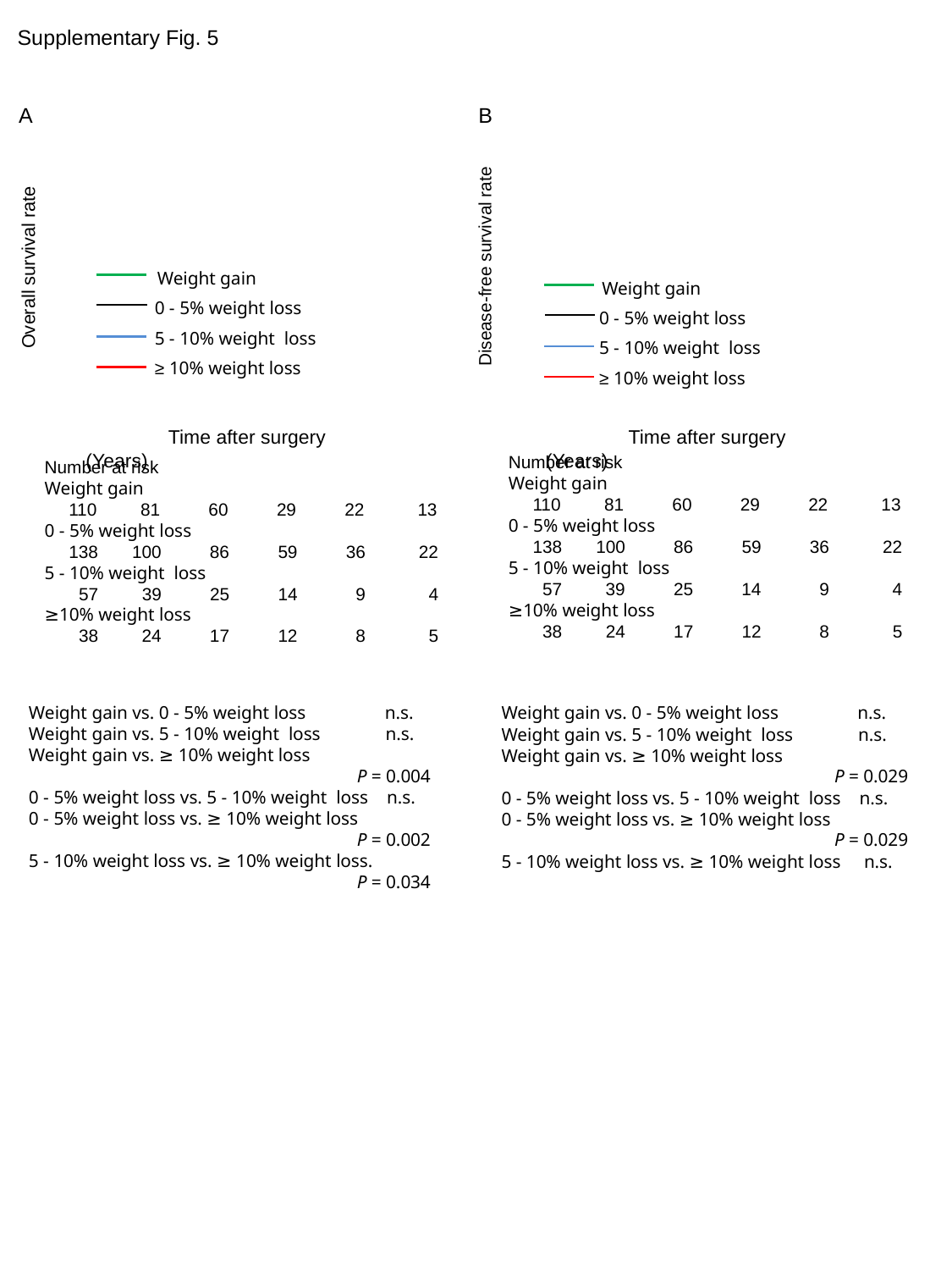

Supplementary Fig. 5
A
B
Disease-free survival rate
Overall survival rate
Weight gain
Weight gain
0 - 5% weight loss
0 - 5% weight loss
5 - 10% weight loss
5 - 10% weight loss
≥ 10% weight loss
≥ 10% weight loss
 Time after surgery (Years)
 Time after surgery (Years)
Number at risk
Weight gain
 110 81 60 29 22 13
0 - 5% weight loss
 138 100 86 59 36 22
5 - 10% weight loss
 57 39 25 14 9 4
≥10% weight loss
 38 24 17 12 8 5
Number at risk
Weight gain
 110 81 60 29 22 13
0 - 5% weight loss
 138 100 86 59 36 22
5 - 10% weight loss
 57 39 25 14 9 4
≥10% weight loss
 38 24 17 12 8 5
Weight gain vs. 0 - 5% weight loss n.s.
Weight gain vs. 5 - 10% weight loss n.s.
Weight gain vs. ≥ 10% weight loss
		 P = 0.004
0 - 5% weight loss vs. 5 - 10% weight loss n.s.
0 - 5% weight loss vs. ≥ 10% weight loss
		 P = 0.002
5 - 10% weight loss vs. ≥ 10% weight loss.
		 P = 0.034
Weight gain vs. 0 - 5% weight loss n.s.
Weight gain vs. 5 - 10% weight loss n.s.
Weight gain vs. ≥ 10% weight loss
		 P = 0.029
0 - 5% weight loss vs. 5 - 10% weight loss n.s.
0 - 5% weight loss vs. ≥ 10% weight loss
		 P = 0.029
5 - 10% weight loss vs. ≥ 10% weight loss n.s.
