# Supplementary material for: Impact of Body Weight Loss During Preoperative Chemoradiotherapy on Prognosis of Patients With Lower Rectal Cancer
Source: Ann Gastroenterol Surg. 2025 Dec 15;10(3):722–38. doi: 10.1002/ags3.70146 (PMC13178290; doi:10.1002/ags3.70146)
Supplement: Supplementary file 2 — Table S1: Relationships between body weight loss and adverse events during CRT. [file AGS3-10-722-s001.docx]

|  | **Supplementary Table 1 – Relationships between body weight loss and adverse events during CRT** | | | | | | | |
| --- | --- | --- | --- | --- | --- | --- | --- | --- |
|  | |  |  |  | Body weight loss  <10% |  | Body weight loss ≥10% |  |
| Toxicity | | | N (%) |  | (n = 302) |  | (n = 41) | *P* value |
| overall | | ≥ grade 3 | 27 (7.9) |  | 20 (6.6) |  | 7 (17.1) | 0.037 |
| Leukopenia | | ≥ grade 3 | 13 (3.8) |  | 9 (3.0) |  | 4 (9.8) | 0.065 |
| Neutropenia | | ≥ grade 3 | 10 (2.9) |  | 6 (2.0) |  | 4 (9.8) | 0.022 |
| Anemia | | ≥ grade 3 | 3 (0.9) |  | 2 (0.7) |  | 1 (2.4) | 0.330 |
| AST abnormality | | ≥ grade 3 | 5 (1.5) |  | 4 (1.3) |  | 1 (2.4) | 0.605 |
| ALT abnormality | | ≥ grade 3 | 6 (1.8) |  | 5 (1.7) |  | 1 (2.4) | 0.732 |
| Anorexia | | ≥ grade 3 | 3 (0.9) |  | 2 (0.7) |  | 1 (2.4) | 0.330 |
| Nausea | | ≥ grade 3 | 3 (0.9) |  | 3 (1.0) |  | 0 (0) | 0.381 |
| Fatigue | | ≥ grade 3 | 3 (0.9) |  | 3 (1.0) |  | 0 (0) | 0.381 |
| Diarrhea | | ≥ grade 3 | 13 (3.8) |  | 9 (3.0) |  | 4 (9.8) | 0.065 |
| Anorectal pain | | ≥ grade 3 | 5 (1.5) |  | 5 (1.7) |  | 0 (0) | 0.257 |
|  | Values in parentheses are percentages, unless indicated otherwise, * Values are medians (interquartile ranges) IQR; Interquartile range, CRT; Chemoradiotherapy, CTCAE; Common Terminology Criteria for Adverse Events | | | | | | | |
